# Correlation of Ki-67 and E-cadherin expression with tumor grade and brain invasion in meningiomas

**DOI:** 10.12669/pjms.42.6.15280

**Published:** 2026-06

**Authors:** Muhammad Hamad Shabir, Adeel Arif, Wajahat Ahmed Khan, Danyal Khan

**Affiliations:** 1 Dr. Muhammad Hamad Shabir, FCPS-II Trainee in Histopathology, Department of Histopathology, Armed Forces Institute of Pathology (AFIP), Punjab, Pakistan; 2 Dr. Adeel Arif, FCPS Histopathology, Department of Histopathology, Armed Forces Institute of Pathology (AFIP), Punjab, Pakistan; 3 Dr. Wajahat Ahmed Khan, FCPS Histopathology, Department of Histopathology, Armed Forces Institute of Pathology (AFIP), Punjab, Pakistan; 4 Dr. Danyal Khan, FCPS-II Trainee in Histopathology, Department of Histopathology, Armed Forces Institute of Pathology (AFIP), Punjab, Pakistan

**Keywords:** CNS Neoplasm, CNS Tumor, E-Cadherin, Invasive meningioma, Meningioma, Meningioma grading, Neoplasm invasiveness, Prognosis, Ki-67

## Abstract

**Objectives:**

Meningioma is most common primary brain tumor. Meningiomas with brain invasion or higher WHO grade behave more aggressively. The objective of this study was to investigate whether Ki-67 and E-cadherin immunohistochemical markers can be utilized to identify high grade tumors bearing potential for brain invasion.

**Methodology:**

It was a comparative cross-sectional study conducted at Armed Forces Institute of Pathology (AFIP), Rawalpindi from 15 April 2025 to 15 November 2025. Eighty-five cases were included in the study. Immunohistochemistry was performed and analyzed for Ki-67 index and E-cadherin expression (Hscore and categorical groups: low/moderate/strong). Immunohistochemistry markers were compared across brain invasion status and WHO grades using nonparametric tests. ROC and bivariable model were used to calculate the discriminative strengths of these biomarkers. Correlations were assessed using Spearman’s method. Chi-square test was employed to identify significance of association.

**Results:**

Ki-67 index was significantly higher in invasive tumors (p < 0.001) and gradually increased with increasing grade of tumor (p < 0.001). Ki-67 showed significant discriminative ability for brain invasion. Categorical analysis of E-cadherin (based on H-score categorical groups) was also significantly associated with brain invasion (p = 0.002) and grade (p < 0.001).

**Conclusions:**

Ki-67 index and E-cadherin categories strongly correlates with tumor potential of brain invasion and higher WHO grades thus aiding in identifying aggressive meningiomas. Categorical E-cadherin reporting enhances its utility over continuous H-scores alone. Thus, even in the absence of glial tissue in biopsy specimen, these biomarkers may help to suggest invasive nature of tumor in appropriate clinical context.

## INTRODUCTION

Meningiomas are the most common primary brain tumors in adults, accounting for 42.6% of all central nervous system tumors.^1^ They develop from arachnoid cap cells in the meninges and vary widely in behavior. Accurate risk assessment is essential because it guides decisions about surgery, radiation, and how closely patients need to be monitored.

The World Health Organization (WHO) CNS grading divides tumors into three grades based on their microscopic features. Grade-I tumors are benign with good outcomes, while Grade-II (atypical) and Grade-III (anaplastic) tumors have higher recurrence rates and poorer survival.^2^ The 2021 WHO update added molecular features to traditional pathology criteria, reflecting advances in our understanding of these tumors.^3^ Importantly, brain invasion remains a key prognostic factor linked to higher recurrence risk, even when other features seem favourable.^4^

Ki-67 labelling index is used as marker of cell proliferation. The literature showed that higher Ki-67 levels are associated with higher grade, more frequent recurrence, and shorter progression-free survival.^5^ Despite this, the discriminative ability of Ki-67 for brain invasion has rarely been quantified.

Beyond proliferation, the ability of tumor cells to invade surrounding tissue is another important factor. Previous studies have reported membranous variable E-cadherin expression in meningioma, suggest its relation to tumor grade and outcomes.^6^ However, this data remains scarce and conflicting as most of the studies pertain to pre-2021 WHO classification of CNS tumors.

In this study, we aimed to evaluate whether Ki-67 and E-cadherin immunohistochemistry may be utilized to access the grade and brain invasiveness of meningioma as an indication of aggressiveness. Moreover, correlation of both of these markers will be studies to predict brain invasion in the absence of morphological evidence in the biopsy. These findings may help to refine risk assessment and guide decisions about follow-up and treatment.

## METHODOLOGY

The study was conducted at Armed Forced Institute of Pathology (AFIP). Sample size was calculated using WHO Sample Size Calculator.^7^ By taking anticipated population proportion of meningioma in Pakistan (P) as 15%^8^, keeping confidence level at 95% (α = 0.05) and acceptable absolute precision (d) at 0.08, minimum sample size came out to be 77. A total of 85 samples were enrolled in the study. Data collection was done from 15 April 2025 to 15 November 2025 followed by statistical analysis, writeup, critical review and editing.

### Ethical approval:

The study was approved by the institutional review board of research, ethics and academic department (READ) of AFIP (Reference: FC-HSP22-22/READ-IRB/23/2562; dated July 3, 2023). Written consent was obtained from the participants.

Immunohistochemistry (IHC) was performed on 4-5 μm thick sections on adhesive slides from formalin-fixed, paraffin-embedded tumor blocks. E-cadherin and Ki-67 immunohistochemistry was applied using Leica biosystems® BOND-III automated IHC and ISH stainer. K2 clone of Ki-67 (Platform: BOND, Lot No. 85888) and clone 36B5 of E-cadherin (Platform: BOND, Lot No. 84661) by Leica biosystems® were utilized for the study. Immunohistochemistry expression was evaluated using conventional immunohistochemical protocols on Olympus® CX23. Brain invasion by meningioma is characterized by “irregular, tongue-like protrusions of tumor cells into underlying brain parenchyma, without intervening leptomeninges” under conventional microscopy.^9^

The Ki-67 labelling index was calculated manually as the percentage of tumor cells nuclei stained in hotspot. Values were subsequently categorized into low (<4%), intermediate (4-19%), and high (≥20%) categories. Expression of E-cadherin was recorded semi-quantitatively using H-score method. The H-score method integrates both staining intensity and the proportion of tumor cells stained:

H-Score= (1 × % Weakly Positive Cells) + (2 × % Moderately Positive Cells) + (3 × %

Strongly Positive Cells)

A total score of 300 was generated using this method. H-Score was further divided into following ordinal categories; Negative (H-score=0), low positive (H-score= 1-100), moderate positive (H-score= 101-200) and strong positivity (H-score 201-300). To reduce the personal bias in calculating Ki-67 and E-cadherin scores, the assessment was independently performed by two histopathologists and mean was calculated for each.

Descriptive statistics were expressed as mean ± standard deviation (SD), median and interquartile range (IQR). Normality of data distribution was estimated using the Shapiro–Wilk test and followed by evaluation of skewness and kurtosis coefficients. IBM® SPSS® Statistics v. 27 was used for statistical analysis.

The Mann–Whitney U test was used to compare continuous variables. Relationships between categorical variables were analyzed using Pearson’s chi-square (χ^2^) test. Caution was practiced for results from contingency tables containing cells with expected counts below five. Multivariable analysis adjusted for WHO grade was not performed in this study as brain invasion is a defining criterion for Grade-II tumors under the WHO 2021 classification.^3^ It resulted creating quasi-complete separation in the model. Univariable logistic regression was implied to assess the ability of the biomarkers to predict brain invasion and bivariable model was used to address potential confounding. ROC was performed to assess discriminative ability of these biomarkers. Spearman’s rank correlation coefficients (ρ) were tested to assess relationships between Ki-67 category, E-cadherin category, WHO grade and brain invasion. Whole of statistical analysis utilized two-tailed statistical tests and a *p*-value <0.05 was considered statistically significant.

## RESULTS

The study included 85 histologically confirmed meningioma cases. Median age of presentation was 52 years (IQR: 15.5 years, Range: 12-75 years). Most of the participants were male (n=49). Most frequently encountered histologic subtype was meningothelial meningioma. The most common grade was WHO Grade-1 contributing to 68 cases (80%), followed by WHO Grade-2 and then WHO Grade-3 (16.5% and 3.5% respectively). Brain parenchymal invasion by the tumor was seen in 14 cases (16.5%).

E-cadherin IHC revealed varying pattern of intensity among the different histological subtypes. Strong membranous E-cadherin immunoreactivity was noted in 41 cases (48.2%), while 39 cases (45.9%) showed moderate intensity and five cases (5.9%) showed low positivity. Average Ki-67 index was 4.36 ± 6.32% (range: 1–35%). The overall median Ki-67 labelling index was 2.0% (IQR: 1.0–4.0%, range 1.0-35.0%) with marked right skew (skewness = 3.09). A stepwise increase in Ki-67 was observed across WHO grades: median 2.0% (IQR: 1.0–3.0%) in Grade-I, 8.0% (IQR: 2.0–16.8%) in Grade-II, and 16.0% (IQR: 15.0–35.0%) in Grade-III (Kruskal-Wallis p < 0.001). Sixty-one cases (71.8%) displayed low proliferation indices, 20 cases (23.5%) demonstrated intermediate proliferative activity, while remaining four cases (4.7%) exhibited high proliferative rates.

Statistical analysis disclosed a significant association between Ki-67 and brain parenchymal invasion. Those tumors which possessed brain parenchymal invasion exhibited high Ki-67 rates as compared to the ones showing no brain invasion (Mann-Whitney test U = 127.5, Z = -4.51, p-value: <0.001). Similarly, Ki-67 category showed a strong association with brain invasion (χ^2^ = 23.93, df = 2, p < 0.001). Majority of the non-invasive tumors were in the low Ki-67 category. When we assessed Ki-67 with tumor grades, a statistically significant association was found (χ^2^ = 28.09, df = 4, p-value < 0.001; Cramér’s V ≈ 0.41). Ki-67 was low in WHO grade I tumors, however it gradually increased from intermediate too high in grade II and grade III tumors, respectively.

E-Cadherin categories displayed significant association both with brain invasion (χ^2^ = 12.13, df = 2, p = 0.002, Cramer’s V = 0.38) and WHO grade (χ^2^ = 19.45, df = 4, p < 0.001; Cramer’s V = 0.34). However, E-cadherin’s continuous H-score did not show significant difference between brain parenchymal invasion (Mann-Whiteny test U = 390, Z = -1.27, p-value = 0.204) and WHO grades (H = 3.72, df = 2, p = 0.156).

Univariable logistic regression showed Ki-67 index was significantly associated with brain invasion (OR = 1.286, 95% CI: 1.116–1.483, p = 0.001) suggesting that each 1% increase in Ki-67 was associated with a 28.6% increase in the odds of brain invasion. E-cadherin H-score did not significantly predicated brain invasion (OR = 0.994, 95% CI: 0.984–1.005, p = 0.291).

Bivariable model was performed including both IHC markers combined, Ki-67 retained its significant association with invasion (OR = 1.289, 95% CI: 1.115–1.491, p = 0.001), while E-cadherin showed no independent contribution (OR = 1.001, 95% CI: 0.988–1.014, p = 0.862). The effect size of Ki-67 was relatively unchanged from that of univariable model. It indicates that there is negligible confounding by E-cadherin.

**Fig.1:**
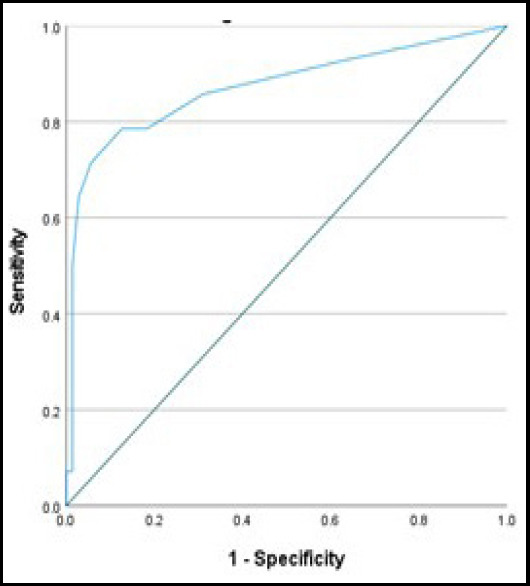
Ki-67 labelling index ROC Curver.

ROC analysis demonstrated that Ki-67 index had significant discriminative ability for brain invasion (AUC = 0.872, 95% CI: 0.746–0.998, p < 0.001) (Fig.1). While, E-cadherin H-score has insignificant and poor discrimination (AUC = 0.608, 95% CI: 0.455–0.761, p = 0.205). The combined model incorporating both biomarkers yielded an AUC of 0.868 (95% CI: 0.730–1.000, p < 0.001), offering no improvement over Ki-67 alone.

Spearman correlation analysis revealed positive correlation of Ki-67 category with brain invasion (ρ = 0.516, p < 0.001) and with WHO CNS grade (ρ = 0.514, p < 0.001). On the other hand, E-Cadherin category revealed inverse correlation with brain invasion (ρ = −0.346, p = 0.001) and tumor grade (ρ = −0.427, p < 0.001). Ki-67 and E-cadherin categories demonstrated a weak inverse correlation (ρ = −0.105, p = 0.341) indicating that higher proliferative activity was linked with lower E-cadherin expression.

## DISCUSSION

In this study, we evaluated immunohistochemical expression of Ki-67 and E-cadherin in 85 cases of meningiomas and analyzed their associations with brain invasion and WHO tumor grade. Most previous studies were conducted under pre-2021 WHO classification and did not confront the analytical challenges posed by invasion being part of grading system. Our study acknowledges these constraints by adopting alternative analytic strategies.

Our study provides comprehensive analysis of both biomarkers with tumor parameters of aggressiveness. Inclusion of histologically confirmed cases of brain invasion enhances the robustness of our findings. The results demonstrate that Ki-67 is significantly higher in both invasive and higher-grade meningiomas and quantified Ki-67 discriminative accuracy for invasion (AUC= 0.872), providing a strong tangible matrix for clinical utility. It also aligns with our observation that Ki-67 was markedly elevated in tumors with brain invasion (Table-I).

**Table-I:** Correlation of Ki-67 categories with invasion

Ki-67 category	Non invasive	Invasive	Invasion rate	95% CI
Low	58	3	4.9%	1.69–13.49
Intermediate	12	8	40.0%	21.88–61.3
High	1	3	75.0%	30.06–95.44

Our study revealed an important methodological observation regarding E-cadherin. We observed that continuous H-scores did not differ significantly by invasion status or WHO grade. This lack of significance reflects inherent limitation for a threshold dependent molecule. E-cadherin mediates cell-cell adhesion in binary manner rather than declining in linear fashion. The continuous H-score dilutes the step-function threshold. This is analogous to categorical and threshold-based scoring of Her2 and PDL1. Therefore, categorical grouping of E-cadherin showed remarkable association with both variables. Low E-cadherin expression was more frequent in invasive and higher-grade tumors and vice-versa for low grade tumors. This finding suggests that simplified categorical reporting may be more clinically relevant to predict brain invasion than continuous scoring for this marker.

Our findings contribute to existing literature by supporting association of high Ki-67 and reduced E-cadherin in aggressive tumors. Most of the studies in literature, in line with our cohort, reported significant increases in Ki-67 labelling index from grade I to grade III tumors indicating its prognostic significance.^10^–^13^ most recently in 2007. Although the latest edition is an improvement over previous grading schemes, the WHO scheme still fails to fully address a variety of important issues regarding the relationship between meningioma histological characteristics and behavior. In particular, routine histological examination fails to identify the subset of Grade I tumors that behave aggressively. Because of this, many additional prognostic markers that require immunohistochemical, cytogenetic, or molecular techniques to evaluate are under investigation. Only one, immunohistochemistry for the proliferation marker, Ki-67 (MIB-1 Demonstration of higher Ki-67 index in invasive meningiomas also aligns with international literature.^14^,^15^ Abry et al.’s systemic review also highlighted Ki-67’s prognostic value but demonstrated remarkable variability in threshold values, likely due to divergent study population and analytical differences.^12^

Various international studies supported our findings of reduced E-cadherin expression with increasing grade and invasiveness of tumors.^16^–^18^ However, some studies suggested unreliability of E-cadherin as an indicator of aggressive meningiomas, possibly due to intratumoral heterogeneity and lack of standardized cutoff values.^19^

### Limitations:

As a single-centered study, it evaluated certain population with similar demography, limiting generalization of its findings. Due to time bound nature of the study and limitation of resources, relatively smaller sample size was included specifically the number of high grade (Grade II/III) / brain invasive meningiomas. This might not accurately represent pattern of Ki-67 and E-cadherin expression in this subset of tumor. Molecular validation could not be performed due to the lack of facility at our institute. Lack of clinical follow up and recurrence data precluded assessment of prognostic significance of these biomarkers.

## CONCLUSION

This study confirms that Ki-67 may be considered a reliable immunomarker of aggressive nature of meningioma. On the other hand, E-cadherin demonstrates prognostic significance only when it was reported categorically rather than as continuous score. Routine assessment of Ki-67 along with categorical analysis of E-cadherin may enhance risk stratification in clinical scenarios where radiological findings fail to demonstrate definitive high-risk features. It will support clinical decision-making regarding follow-up and surveillance intensity in addition to planning surgical treatment.

### Recommendations:

In future, large-scale multicentric studies, incorporating long-term outcomes, may be required with larger proportion of high grade and invasive tumors to confidently assess association of these biomarkers with parameters of interest.

### Authors` Contribution:

**MHS:** Acquisition of data, formal statistical analysis, writing original draft, Initial slides viewing, Acquisition of review board certificate

**AA:** Funding acquisition, Project administration, Supervision, Validation and Resources

**WAK:** Methodology and Investigation, Statistical analysis and responsible for accuracy of study

**DK:** Immunohistochemistry analysis, Visualization (figure and table), Writing – critical review & editing, Literature Review

All authors are agreed to be accountable for all aspects of the work. They have read and approved the final manuscript.
